# Intrapericardial diaphragmatic hernia in a 6-month-old girl: A case report and review of the literature

**DOI:** 10.1016/j.ijscr.2019.05.049

**Published:** 2019-06-05

**Authors:** Matchecane Cossa, Tyler D. Robinson

**Affiliations:** a Departmento de Cirurgia, Hospital Central de Maputo, Maputo, Mozambique; b Ministério de Saúde de Moçambique, Maputo, Mozambique; c Albany Medical Center, Albany, New York, USA

**Keywords:** CDH, congenital diaphragmatic hernia, IPCDH, intrapericardial congenital diaphragmatic hernia, Pericardium, Congenital diaphragmatic hernia, Pediatric surgery, Thoracic surgery, Case report

## Abstract

**Introduction:**

Intrapericardial congenital diaphragmatic hernia (IPCDH) is extremely rare: only 18 cases in children have been reported in the literature since 1981. We report the 19th case of infantile IPCDH in a 6-month-old girl and compare with previous reports.

**Presentation of case:**

Our patient initially presented with four days of dyspnea and was diagnosed with CDH by preoperative radiography. She was taken to the operating room, where an IPCDH was discovered, and successfully reduced.

**Discussion:**

CDH may present with cardiopulmonary compromise, but IPCDH directly compress the heart, leading to acute or subacute heart failure.

**Conclusion:**

Pediatric surgeons should be aware of and prepared for the possibility of IPCDH when making surgical plans to correct CDH.

## Highlights

• Intrapericardial congenital diaphragmatic hernia (IPCDH) is extremely rare.
• We report the 19th case of pediatric IPCDH in a 6-month-old girl.
• Our case of IPCDH was successfully surgically reduced with no significant perioperative complications.
• Pediatric surgeons should be aware of IPCDH.

## 1 Introduction

Congenital diaphragmatic hernia (CDH) is uncommon and intrapericardial congenital diaphragmatic hernia (IPCDH) is extremely rare. The incidence of CDH is estimated to be one per 2200 births [1]. The two primary forms of CDH are: (1) the Bochdalek diaphragmatic hernia, a defect of the postero-lateral diaphragm comprising roughly 95% of CDHs, and (2) the Morgagni diaphragmatic hernia, located in the right-anterior position. IPCDH represents a developmental failure of the retro-sternal portion of the *septum transversum* [2,3]. Here we describe a case of IPCDH with successful surgical repair, and also review the literature including 18 previously described cases of infantile IPCDH. This work is reported in line with the consensus SCARE criteria for case reports [4].

## 2 Case report

A 6-month-old girl presented to the pediatric emergency department with four days of intermittent fever, tachypnea, and persistent cough. According to the patient’s mother, she had not experienced previous nausea, vomiting, dyspnea, or peripheral edema; but she did exhibit poor oral intake and general failure to thrive. Her mother reported no history of trauma, birth complications, or evidence of other congenital malformations and a thorough review of systems was otherwise negative. Her mother was a 23-year-old woman who denied any personal or family history of chronic disease, sick contacts (including anyone infected with tuberculosis), and reported a negative HIV status. She was seen by a physician prenatally but did not receive routine ultrasound.

**Fig. 1:**
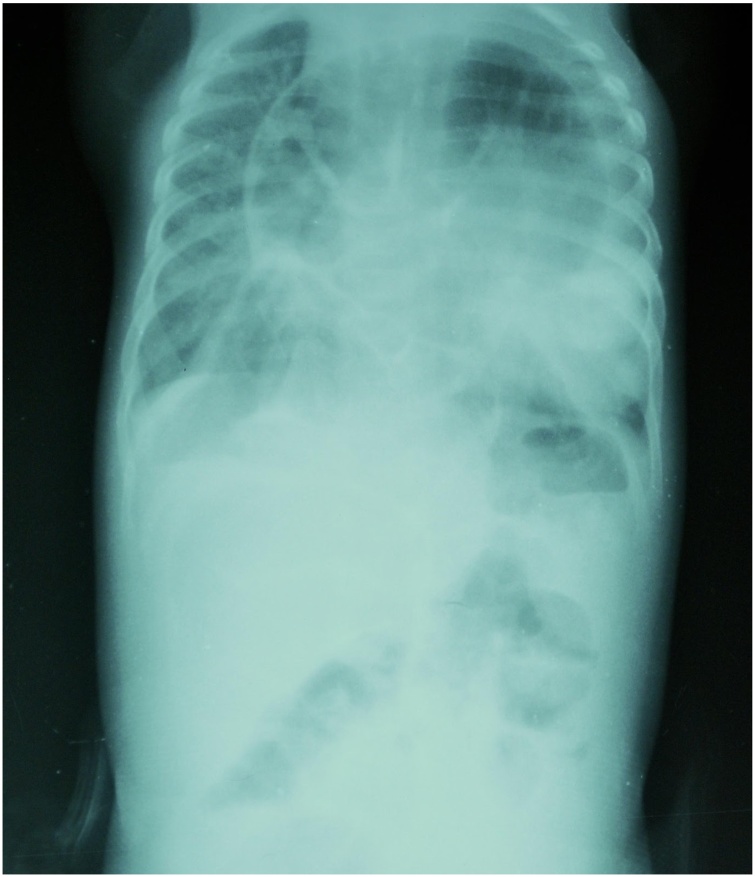
Preoperative chest radiograph clearly demonstrating mediastinal loops of bowel.

**Fig. 2:**
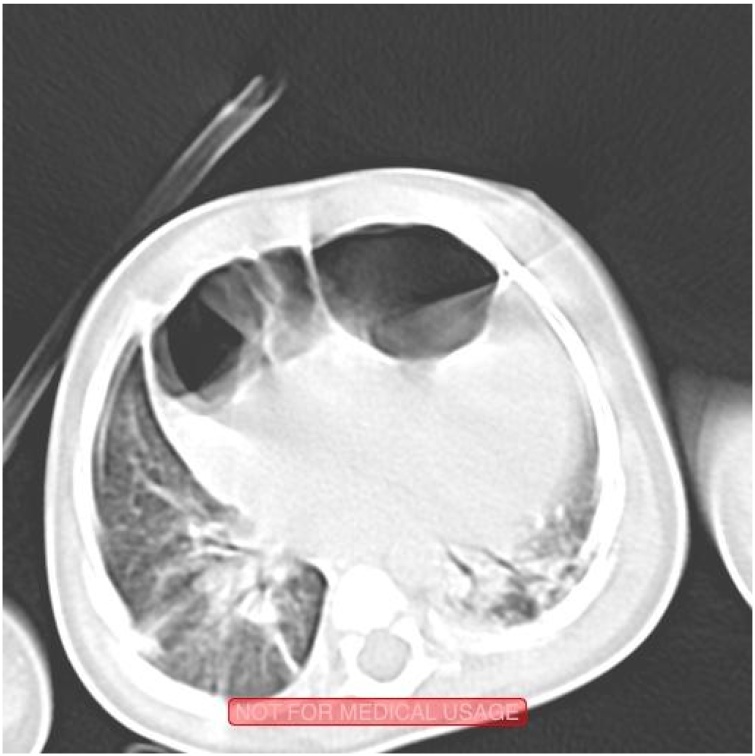
A chest computed tomography scan demonstrating mediastinal loops of bowel.

Physical examination revealed a small female infant with low body weight (4.8 kg, <5 percentile weight for age), breathing at a rate of 52 breaths/min, with a heart rate of 145 beats/min, and blood pressure of 126/111 mmHg. She was afebrile. Abdominal evaluation was soft and nontender. Chest auscultation was notable for diffuse bilateral rhonchi and rales, though cardiac sounds were normal. She was not cyanotic. Preoperative labs demonstrated anemia (HgB: 10.2 g/dL) but no leukocytosis (WBC: 9.4 × 10^3^/μL). Echocardiography was reported as normal. An initial chest radiograph (Fig. 1) showed dilated loops of bowel located centrally in the thoracic cavity, obscuring the cardiac silhouette, with opacification of the lower lobe of the left lung. These findings were suspicious for CDH, which was confirmed by a thoracic CT scan (Fig. 2). Plans for corrective surgery were made with the preoperative diagnosis of a likely Morgagni-type CDH.

**Fig. 3:**
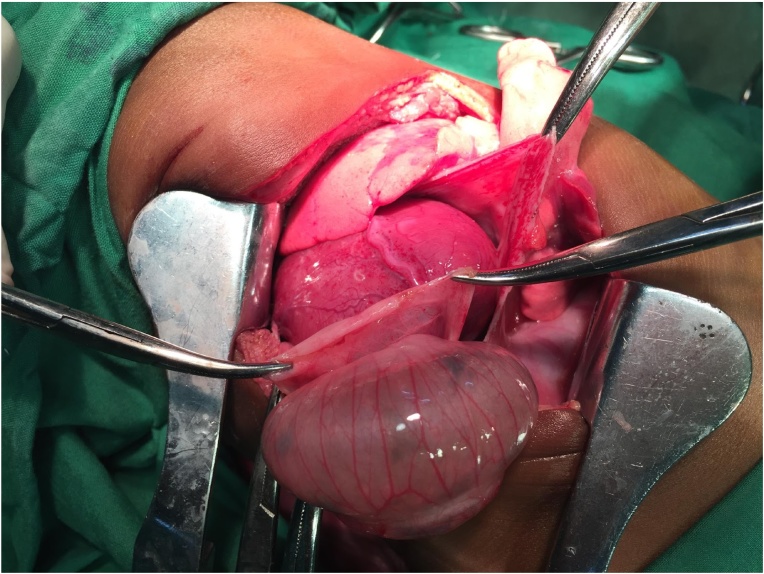
Intraoperative reduction of herniated bowel from within the pericardium.

The patient was taken to the operating room and underwent a left thoracotomy. It was readily apparent that there was no hernia sac or loops of bowel within either pleural cavities. However, the pericardium was grossly enlarged and ballotable. We carefully incised the pericardium, from which drained roughly 20 ml of serous fluid. Within the pericardium we found the hernia sac, which was also incised, revealing loops of small bowel that were incarcerated, but not strangulated (Fig. 3). The heart itself was visualized directly without overlying pericardium. On two occasions during manipulation of the hernia contents the heart became asystolic. Each time systole was quickly restored with direct cardiac massage. We proceeded to reduce 10 cm of small bowel, and corrected the 2 cm defect in the cardiac portion of the central tendon of the diaphragm. The remainder of the diaphragm was intact, the abdominal wall was intact, the great vessels entering and leaving the heart were grossly normal, and we found no evidence of any other congenital malformations within the thorax. We closed the pericardium, leaving a small defect to prevent future pericardial effusion. We subsequently closed the thoracic cavity in layers, leaving a 28 French chest tube draining the pleural cavity by gravity under water seal.

Postoperative management included treatment with furosemide for acute pulmonary edema on post-operative day one. On the second post-operative day, the patient developed a new leukocytosis and fever. A chest radiograph demonstrated bilateral opacification of the lung fields. She was given penicillin, acetaminophen, and salbutamol and a follow-up chest radiograph demonstrated clear lung fields and no evidence of hernia. The patient was discharged seven days after her operation, breastfeeding normally clinically stable. Despite attempts to contact the mother, the patient was lost to follow up and not seen in our surgical clinic.

## 3 Discussion

The abdominal wall, sternum, pericardium, and part of the diaphragm arise from somatopleuric layer of lateral plate mesoderm. Errors in embryogenesis of these structures during weeks 4–10 give rise to various malformations, most commonly omphalocele and gastroschisis. A study of 362 patients with diagnosed CDH found 39.5% of cases to have associated congenital malformations [5]. Though the frequency of additional congenital malformations associated with IPCDH has not been determined, malformations such as omphalocele [6], low-set ears, facial nerve palsy, choanal atresia, and eye abnormalities [7] have been described in case reports. Our patient had no other congenital anomalies.

IPCDH is a very rare condition, and descriptions of previous cases are variable. Since 1981, there have been 22 cases of isolated IPCDH reported in the literature: 18 cases in infants, one in a child, and three cases in adults. Of the 18 infant cases, eight were diagnosed prenatally by ultrasound, seven diagnosed during the first three days of life, and three diagnosed at one week to six months of life [2,3,8,9]. One case of IPCDH was not diagnosed until five years of age [10]. Other cases have been diagnosed in mid [11,12] or late [13] adulthood. The most common cause of diaphragmatic hernia in adults is trauma, and it is unclear if adult cases share a similar pathophysiology as the congenital cases found in infants.

The presentation of IPCDH is diverse [10], with varying symptomatic severity and cardiopulmonary compromise. In patients whose hernias were diagnosed by prenatal ultrasound, corrective surgery was performed within hours of induced delivery, precluding the development of symptoms [3,8]. In a neonate without a prenatal CDH diagnosis, symptoms developed on day two of life, and included prandial tachypnea and cyanosis [6]. A 5-year-old female presented with a one-year history of easy fatigability and dyspnea on exertion [10]. In our case, the patient was below the fifth percentile of weight-for-age and demonstrated failure to thrive. She was tachypneic and coughing at presentation, but was not acutely ill and, per her mother’s reports, had never exhibited symptoms of acute distress prior to presentation. One other case of IPCDH in a 3-month-old infant presented similarly, with persistent cough, tachypnea, and failure to thrive [9].

In all cases reported, the herniated bowel was healthy and successfully reduced into the abdomen with primary closure or with mesh closure of the diaphragmatic defect. There are three IPCDH-associated deaths reported: (1) two days after corrective surgery in a neonate; (2) three hours after birth and without surgical intervention; and (3) in termination of pregnancy [2]. All other patients had successful outcomes without report of any complications. There is no data on long-term health outcomes of patients with surgically repaired IPCDH.

The diagnosis of all CDHs relies primarily on radiography. Bochdalek-type CDH presents on chest radiography with intra-thoracic viscera, with 80–90% of hernias on the left side [14]. Chest radiography of the rarer Morgagni-type CDH includes intra-thoracic opacification or obvious viscera, the large majority on the right side [15]. All suspected CDH are confirmed with computed tomography. We and others [6,9,10] have found that diagnosis of IPCDH is often made intraoperatively. This is likely due to the rarity of the condition, which is radiographically similar to Morgagni-type CDH, with hernia contents present near the heart. IPCDH can present with pericardial effusion [2,8], which can obscure the loops of bowel and delay diagnosis. However, as can be noted from Fig. 1, chest radiography of IPCDH demonstrates elliptical, retrosternal loops of bowel, which may radiographically distinguish IPCDH from other types of CDH.

Surgical management of IPCDH requires pericardial incision, reduction of hernia contents through the diaphragmatic defect, and closure of the defects. Although we chose to leave the pericardium only partially closed, a review article of 240 publications of cardiac surgery concluded that there is no evidence of adverse clinical outcomes in adults when the pericardium is closed completely after pericardiotomy [16].

Although very rare, IPCDH is a diagnosis to consider when managing children with suspected CDH. With prompt surgical intervention, most patients have a good recovery and prognosis.

## Conflicts of interest

No conflicts of interest to disclose.

## Sources of funding

There was no external funding obtained to support the creation of this manuscript.

## Ethical approval

This case report is exempt from ethical approval per institutional policy.

## Consent

Written informed consent was unable to obtained from the patient for publication of this case report and accompanying images. The authors made exhaustive attempts to contact the patient, which were not successful. In our hospital’s estimation, no patient will be harmed due to the publication of this deidentified case report. A letter from a senior hospital administrator is available for review from the Editor-in-Chief of this journal on request.

## Author contribution

Matchecane Cossa: Conceptualization; Formal analysis; Supervision; Writing - review & editing.

Tyler Robinson: Methodology; Roles/Writing - original draft.

All authors approved the final version of this manuscript.

## Registration of research studies

As this is a case report and not an interventional study, this manuscript is exempt from registration.

## Guarantor

Tyler Robinson, MD, MPH.

## Provenance and peer review

Not commissioned, externally peer-reviewed.
